# Association between polymorphisms in the promoter region of *miR‐17‐92* cluster and systemic lupus erythematosus in a Chinese population

**DOI:** 10.1111/jcmm.13672

**Published:** 2018-05-16

**Authors:** Rong Wang, Chun‐Fang Wang, Hai‐Mei Qin, Yu‐Lan Lu, Gui‐Jiang Wei, Hua‐Tuo Huang, Yang Xiang, Jun‐Li Wang, Yan Lan, Ye‐Sheng Wei

**Affiliations:** ^1^ Youjiang Medical University for Nationalities Baise China; ^2^ Department of Laboratory Medicine The Affiliated Hospital of Youjiang Medical University for Nationalities Baise China; ^3^ Department of Dermatology The Affiliated Hospital of Youjiang Medical University for Nationalities Baise China

**Keywords:** case‐control study, Chinese, *miR‐17‐92*, polymorphism, systemic lupus erythematosus

## Abstract

The aim of this study was to investigate the association of genetic polymorphisms in the promoter region of *miR‐17‐92* with systemic lupus erythematosus (SLE). The gene polymorphism was analysed using SNaPshot in 312 SLE patients and 396 controls. Relative expression of miR‐17‐92 was measured by quantitative real‐time PCR. Association was found between rs9515692 and a decreased risk of SLE (CT vs CC: OR = 0.65, 95%CI, 0.46‐0.92, *P* = .014; CT+TT vs CC: OR = 0.64, 95%CI, 0.46‐0.90, *P* = .009; T vs C: OR = 0.69, 95%CI, 0.52‐0.92, *P* = .010, respectively). Haplotype analysis showed that C‐G‐G, C‐A‐A haplotypes were associated with an increased SLE risk (OR=4.46, 95%CI, 2.17‐9.17, *P* < 0.001; OR=2.33, 95%CI, 1.44‐3.76, *P* < 0.001, respectively). T allele and CT+TT genotypes in rs9515692 were associated with decreased risk of anti‐dsDNA in SLE (CT+TT vs CC: OR = 0.42, 95%CI = 0.24‐0.72, *P* = .002; T vs A: OR = 0.49, 95%CI = 0.31‐0.79, *P* = .003). Moreover, rs9515692 CT+TT genotypes had a higher level of miR‐17 as compared to CC genotype (*P* = .017). These findings suggest that the rs9515692 CT+TT genotypes were a protective factor for the susceptibility of SLE, probably by increasing the expression of miR‐17.

## 1 INTRODUCTION

Systemic lupus erythematosus (SLE) is a chronic autoimmune disorder disease characterized by breakdown of tolerance to self‐antigens and production of multiple autoantibodies and immune complexes.^1^ SLE has higher morbidity, disability and mortality rates. Thus, both the quality of life and life expectancy in patients are seriously influenced. In China, the prevalence rate of SLE is about 0.03%: higher than in Japan, or Europe and America.^2^ The aetiology of SLE has not been fully elucidated clearly. Recent studies showed that genetic factors might be associated with the individual susceptibility to SLE. Previous studies have been established that genes have important effects on the development of SLE such as *LEP* and *LEPR* gene, *Fc*γ*R* gene, miRNA‐146a.^3^, ^4^, ^5^ Successes from these studies generally explain only part of disease heritability in SLE. Hence, the molecular genetic basis of this disease remained deficiently understood.

MiR‐17‐92 displays different expression level during B‐cell development: they are enhanced level in progenitor cells, and their expression decreases highly when pre‐B becomes immature B cells.^6^ MiR‐17‐92 expression is up‐regulated in CD4^+^ T cells from lupus patients and multiple sclerosis.^7^, ^8^ Decreased expression of miR‐17‐92 has been found during differentiation towards CD8^+^ T cells.^9^ It is generally recognized that these immune‐related cells were related to the development of SLE.

To date, no report was carried out to investigate the association of SNPs in *miR‐17‐92* and SLE risk, and the relationship between *miR‐17‐92* gene SNPs and the expression of plasma miR‐17‐92 family members in SLE patients. We use EPD (http://www.epd.isb-sib.ch/) and miRbase (http://www.mirbase.org/) to predictive promoter. Then, the SNPs of Minor allele frequency (MAF) greater than 10% were selected by UCSC (http://genome.ucsc.edu) in the Chinese population. Therefore, we evaluate the association of the three SNPs (rs9515692 and rs1352743) in the promoter region of *miR‐17‐92* with susceptibility to SLE and further investigate the influence of *miR‐17‐92* polymorphisms on critical plasma levels and various disease clinical features.

## 2 MATERIALS AND METHODS

### 2.1 Subjects of study

The study comprised 312 patients (62 males and 250 females) with SLE diagnosed according to the American College of Rheumatology classification (ACR) 1997 criteria for SLE. At the same time, 396 ethnically matched healthy controls (98 males and 298 females) without any history of autoimmune disease, inflammatory and chronic infectious diseases were selected. The clinical characteristics of patients and controls are shown in Table S1. The patients and healthy individuals were selected at the Affiliated Hospital of Youjiang Medical University for Nationalities, Baise, China. The Ethics Committees of Affiliated Hospital of Youjiang Medical University for Nationalities approved this study protocol. The study was carried out in accordance with the relevant guidelines. Written informed consent was obtained from all the participants.

### 2.2 DNA extraction and miR‐17‐92 genotyping

EPD (http://epd.vital-it.ch/index.php) and miRbase (http://www.mirbase.org/) were used to predictive promoter. We conducted a search for the *miR‐17‐92* gene single nucleotide polymorphisms (SNPs) with a minor allele frequency (MAF) ≥ 10% and label SNPs within the Han Chinese population (CHB) of Beijing, China. Genomic DNA was extracted using whole‐blood genome DNA extraction kit (Tiangen Inc., Beijing, China). The designs of PCR primers were carried out by online primer 3.0 software (http://primer3.ut.ee/). SNaPshot was used to analyse genotypes of SNPs. The PCR primers are shown in Table S2.

### 2.3 Quantitative PCR of miR‐17‐92

Total RNA was isolated from 100 μL plasma performed with a commercial kit (Takara, Dalian, China) following the manufacturer's protocol. Five microgram of total RNA was transcribed into cDNA utilizing Mir‐X miRNA First‐Strand Synthesis Kit (Takara, Cat. No. 638315). Quantitative PCR was performed using Mir‐X miRNA qRT‐PCR SYBR Kit (Takara, Cat. No.638313) and ABI 7900HT real‐time PCR machine (Applied Biosystems, CA, USA). The 3^′^ primers for quantitative PCR are mRQ 3^′^ Primer supplied with the kit. Cel‐miR‐39 was used as an internal control. Relative expression levels of miR‐17‐92 were calculated using the delta‐delta Ct method (2^−ΔΔCt^).

### 2.4 Statistical analysis

If the data were normally distributed variables, the Student's t‐test was used; otherwise, Mann‐Whitney U test was used. Hardy‐Weinberg equilibrium (HWE) was tested using chi‐squared test. Haplotype analysis was performed using SHEsis software (http://analysis.bio-x.cn/myAnalysis.php) . OR and 95% CI were adjusted based on age and gender using logistic regression. *P* < .05 was considered statistically significant.

## 3 RESULTS

There was no significant difference between cases and controls in age (*P* = .087) and gender (*P* = .124). The distributions of the four SNPs polymorphisms in SLE and controls are shown in Table 1. All genotype distributions were in agreement with the Hardy‐Weinberg equilibrium of any SNP (*P* > .05). To note, the CT genotype and dominant model (CT+TT) in rs9515692 were associated with decreased risk of SLE (CT vs CC: AOR = 0.65, 95% CI, 0.46‐0.92, A*P* = .014; CT+TT vs CC: AOR = 0.64, 95% CI, 0.46‐0.90, A*P* = .009). In addition, T allele was associated with decreased risk of SLE (T vs C: AOR = 0.69, 95% CI, 0.52‐0.92, A*P* = .010). Nevertheless, no significant association between the remainder two SNPs and SLE was observed (A*P* > .05).

**Table 1:** Genotype and allele distributions of three SNPs in *miR‐17‐92* in SLE and control groups

Polymorphisms	SLE (%)	Controls (%)	OR (95% CI)	AOR (95% CI)^a^	*P*	*AP* a
rs9515692						
CC	234 (75.0)	260 (65.7)	1.00 (Ref)			
CT	68 (21.8)	118 (29.8)	0.64 (0.45‐0.91)	0.65 (0.46‐0.92)	.006	.014
TT	10 (3.2)	18 (4.5)	0.62 (0.28‐1.36)	0.62 (0.28‐1.37)	.229	.235
Dominant model	78 (25.0)	136 (34.3)	0.64 (0.46‐0.89)	0.64 (0.46‐0.90)	.007	.009
Recessive model	302 (96.8)	378 (95.5)	1.44 (0.65‐3.16)	1.44 (0.65‐3.18)	.364	.364
C	536 (85.9)	638 (80.6)	1.00 (Ref)			
T	88 (14.1)	154 (52.4)	0.68 (0.51‐0.91)	0.69 (0.52‐0.92)	.008	.010
rs1352743						
AA	41 (13.1)	51 (12.9)	1.00 (Ref)			
AG	164 (52.6)	193 (48.7)	1.06 (0.67‐1.68)	1.08 (0.68‐1.71)	.814	.756
GG	107 (34.3)	152 (38.4)	0.88 (0.54‐1.42)	0.88 (0.55‐1.43)	.587	.609
Dominant model	171 (86.9)	345 (87.1)	0.98 (0.63‐1.52)	0.99 (0.64‐1.54)	.918	.964
Recessive model	205 (65.7)	244 (61.6)	1.20 (0.88‐1.63)	1.20 (0.88‐1.64)	.262	.245
A	246 (39.4)	295 (37.2)	1.00 (Ref)			
G	378 (60.6)	497 (62.8)	0.91 (0.74‐1.13)	0.91 (0.73‐1.13)	.912	.402
rs1813389						
AA	124 (39.7)	164 (41.4)	1.00 (Ref)			
AG	157 (50.4)	194 (49.0)	1.07 (0.78‐1.46)	1.08 (0.79‐1.49)	.671	.615
GG	31 (9.9)	38 (9.6)	1.08 (0.64‐1.83)	1.07 (0.63‐1.81)	.778	.817
Dominant model	188 (60.3)	232 (58.6)	1.07 (0.79‐1.45)	1.08 (0.80‐1.47)	.653	.615
Recessive model	281 (90.1)	358 (90.4)	0.96 (0.58‐1.59)	0.98 (0.59‐1.62)	.880	.943
A	405 (64.9)	522 (65.9)	1.00 (Ref)			
G	219 (35.1)	270 (34.1)	1.05 (0.84‐1.30)	1.05 (0.84‐1.31)	.693	.690

a Adjusted by age and gender.

miRNA, microRNA; SLE, systemic lupus erythematosus; 95% CI, 95% confidence interval; AOR, adjusted OR value; A*P*, adjusted *P* value; Ref, reference.

Haplotype analysis was performed by online SHEsis software and the three haplotypes (C‐G‐G, C‐A‐A) were associated with increased risk of SLE (OR = 4.46, 95% CI, 2.17‐9.17, *P* < .001; OR = 2.33, 95% CI, 1.44‐3.76, *P* < .001, respectively).

**Figure 1:**
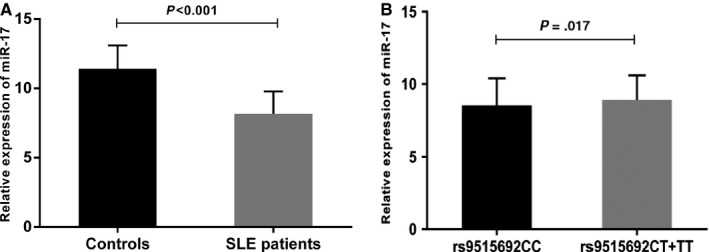
Relative expression of miR‐17 in SLE patients (n = 96) and healthy controls (n = 96). A, Decreased level of miR‐17 in SLE patients compared with healthy controls (*P* < .001). B, Increased level of miR‐17 in SLE patients carrying the rs9515692 CT+TT compared with those carrying the rs9515692CC (*P* = .017). Data are presented as mean ± standard error. Expression levels were normalized to cel‐miR‐39

Additionally, the expression of miR‐17 in SLE patients was decreased significantly compared with control subject (*P* < .001; Figure 1A). After confirming the genetic association of rs9515692 with SLE susceptibility, we aimed to analyse if this SNP would have an effect on expression levels on miR‐17‐92. We found that the rs9515692 CT/TT had a higher level of miR‐17 compared with those carrying the rs9515692 CC (*P* = .017; Figure 1B).

We compared the distribution of genotypic frequencies of rs9515692 between positive and negative patients in thirteen specific clinical manifestations and found that rs9515692 CT+TT and T allele with anti‐dsDNA in distribution of allele and genotype frequencies (CT+TT vs CC: AOR = 0.42, 95% CI = 0.24‐0.72, A*P* = .002; T vs A: AOR = 0.49, 95% CI = 0.31‐0.79, A*P* = .003) (Table 2).

**Table 2:** Association of allele and genotype frequencies in rs9515692 with clinical features in SLE

Clinical features	Allele [n]		AOR^a^	*AP* a	Genotype [n]		AOR^a^	*AP* a
	C	T	T vs C		CC	CT+TT	CT+TT vs CC	
Malar rash								
Positive	161	27	1.05 (0.64‐1.71)	.850	69	25	1.16 (0.67‐2.03)	.595
Negative	375	61			165	53		
Photosensitivity								
Positive	302	48	0.92 (0.58‐1.45)	.717	132	43	0.94 (0.56‐1.58)	.814
Negative	234	40			102	35		
Leucopenia								
Positive	334	54	0.96 (0.60‐1.53)	.865	147	47	0.90 (0.53‐1.52)	.684
Negative	202	34			87	31		
Anaemia								
Positive	291	45	0.85 (0.54‐1.33)	.471	126	42	0.94 (0.56‐1.58)	.808
Negative	245	43			108	36		
Complement depressed								
Positive	377	59	0.86 (0.53‐1.39)	.531	165	53	0.88 (0.51‐1.54)	.658
Negative	159	29			69	25		
Renal disorder								
Positive	284	38	0.68 (0.43‐1.07)	.097	127	34	0.66 (0.39‐1.11)	.114
Negative	252	50			107	44		
Neurologic disorder								
Positive	121	23	1.24 (0.74‐2.09)	.415	51	21	1.38 (0.76‐2.51)	.286
Negative	415	65			183	57		
Arthritis								
Positive	324	50	0.89 (0.56‐1.42)	.627	141	46	1.02 (0.60‐1.73)	.952
Negative	212	38			93	32		
Anti‐dsDNA								
Positive	272	30	0.49 (0.31‐0.79)	.003	125	26	0.42 (0.24‐0.72)	.002
Negative	264	58			109	52		
Anti‐RNP								
Positive	220	30	0.74 (0.46‐1.19)	.210	98	27	0.73 (0.42‐1.24)	.242
Negative	316	58			136	51		
Anti‐Sm								
Positive	211	37	1.14 (0.72‐1.81)	.576	91	33	1.20 (0.71‐2.02)	.504
Negative	325	51			143	45		
Anti‐SSA								
Positive	355	63	1.26 (0.77‐2.08)	.359	153	56	1.31 (0.74‐2.30)	.355
Negative	181	25			81	22		
Anti‐SSB								
Positive	121	27	1.51 (0.92‐2.49)	.103	50	24	1.64 (0.92‐2.92)	.095
Negative	415	61			184	54		

a Adjusted by age and gender.

95% CI, 95% confidence interval; AOR, adjusted OR value; A*P*, adjusted *P* value; n, number.

## 4 DISCUSSION

The majority of studies have followed with interest on miRNA expression levels in diseases. Variations in the miRNA genes could contribute to abnormal secretion of miRNAs and even could be linked with susceptibility of diseases. A few studies have investigated the relationship between SNPs in miRNA regions and SLE. For example, Lofgren et al^5^ found that rs2431697 was not associated with expression levels of PTTG1, but with the miR‐146a, and the risk allele had lower expression of the miRNA. The above results indicated that SNP is potentially important in SLE aetiology. Changes in the miR‐17 expression have been observed in more human diseases, such as inflammatory and autoimmune diseases, viral infections and cancer.^10^, ^11^, ^12^ Carlsen et al^13^ demonstrated that miR‐17 expression was decreased significantly in plasma of SLE patients. Consistent with the result, we found that decreased level of miR‐17 in SLE patients was compared with healthy controls. This indicated that miR‐17 expression in plasma may suppress SLE development. One of the most characteristic antibodies of SLE is anti‐double‐stranded DNA (Anti‐dsDNA), which as a sensitive symbol in the disease. Overexpression of miRNA17‐92 cluster was associated high titres of anti‐DNA antibodies.^8^ We identified that rs9515692 was related to decreased risk of anti‐dsDNA. The finding further provides evidence that rs9515692 may be a protective factor for SLE. The molecular mechanism of how the rs9515692 leads to the up‐regulation of miR‐17 requires a more detailed analysis.

MiR‐17‐92 is strongly induced in activated T cells. Both mRNA and protein level of E2F1 were significantly decreased in SLE patients, miR‐17 of miR‐17‐92 cluster is critically involved in Th17 differentiation. Not only that, the miR‐17‐92 cluster also is an important mediator of Tfh cell biology. Deletion of the miR‐17‐92 cluster brought about decrease in numbers in Tfh and germinal centre of B cells, while transgenic expression of this miRNA cluster in CD4^+^ T cells caused increased numbers of both Tfh and germinal centre B cells.^14^, ^15^ Decreased expression of pro‐apoptotic molecule and phosphatase and tensin homologue on chromosome 10 in mice transgenic for the miR‐17‐92 cluster, lead to lymphoproliferation and other lupus manifestations.^16^ It is generally recognized that these cells play primary mediators in SLE. Given the key roles of miR‐17‐92 in SLE development, the positive results in our present study were biologically reasonable.

In conclusion, this is the first time reported *miR‐17‐92* gene polymorphisms associated with SLE susceptibility in an independent Chinese cohort. Analyses suggested that the rs9515692 decreased the risk of SLE in the Chinese population. Therefore, our findings may provide new insights into the development of SLE and create an opportunity to approach the diagnosis and treatment. In the future, further functional studies of rs9515692 will help us to define the potential biological mechanism of SLE.

## CONFLICT OF INTERESTS

The authors confirm that there are no conflict of interests.
