# Supplementary material for: Association between polymorphisms in the promoter region of miR‐17‐92 cluster and systemic lupus erythematosus in a Chinese population
Source: J Cell Mol Med. 2018 May 16;22(8):4016–20. doi: 10.1111/jcmm.13672 (PMC6050484; doi:10.1111/jcmm.13672)
Supplement: Supplementary file 1 [file JCMM-22-4016-s001.docx]

| **Table S1 Clinical characteristics of the SLE patients and controls.** | | | |
| --- | --- | --- | --- |
| **Characteristics** | **SLE**  **n=312 (%)** | **Controls**  **n=396 (%)** | ***P*** |
| Age(median, years) | 37.86±13.01 | 39.48±12.10 | 0.087 |
| Male/Female | 62(19.9)/250(80.1) | 98(24.7)/298(75.3) | 0.124 |
| Malar rash | 94(30.1) | - | - |
| Photosensitivity | 175(56.1) | - | - |
| Leucopenia | 194(62.2) | - | - |
| Anemia | 168(53.8) | - | - |
| Complement depressed | 218(69.9) | - | - |
| Renal disorder | 161(51.6) | - | - |
| Neurologic disorder | 72(23.1) | - | - |
| Arthritis | 187(59.9) | - | - |
| Anti-dsDNA | 151(48.4) | - | - |
| Anti-RNP | 125(40.1) | - | - |
| Anti-Sm | 124(39.7) | - | - |
| Anti-SSA | 209(70.0) | - | - |
| Anti-SSB | 69(23.7) | - | - |

| **Table S2 The primer sequences for *miR-17-92* genotyping and RT-PCR** | |
| --- | --- |
| **Item** | **PCR primers** |
| rs9515692 | Upstream primer: 5^′^-GGAAACAACCTGGAGGCTCTTCAA-3^′^ |
|  | Downstream primer: 5^′^-TTTCCTCTAACCTGAACCCCTGTCT-3^′^ |
|  | Extension primer: 5^′^-TTTTTTTTTTTTTTCCAGTGATTTTCC  TTATTACTGCTGA-3^′^ |
| rs1352743 | Upstream primer: 5^′^-TTGCTGACCGTAATCAGCCACAT-3^′^ |
|  | Downstream primer: 5^′^-GGCAAACGACCACAGAGGAAAT-3^′^ |
|  | Extension primer: 5^′^-TTTTTTTTTTTTTTTTTTTTTTTTTTTT  CCTCATTATTTTTAGTAAAAGGGTTGA-3^′^ |
| rs1813389 | Upstream primer: 5^′^-TCGGGGGAGAATAAACGAGAGG -3^′^ |
|  | Downstream primer: 5^′^-TCGCCAGTGCATTAAGCCCTAC-3^′^ |
|  | Extension primer: 5^′^-TTTTTTTTTTTTTTTTTTTTTTTTTTT  CAGCCAGAGTGGATCAACCTT-3^′^ |
| miR-17 | Upstream primer: 5^′^- CAAAGUGCUUACAGUGCAGGUAGC -3^′^ |
| miR-20a | Upstream primer: 5^′^-GCGGCGGTAAAGTGCTTATAGTG-3^′^ |
| miR-18a | Upstream primer: 5^′^-TAAGGTGCATCTAGTGCAGATAG-3^′^ |
| miR-19a | Upstream primer:5^′^-AGUUUUGCAUAGAUUGCACUUCA-3^′^ |
| miR-19b | Upstream primer:5^′^-UGUGCAAAUGCAUGCAAAACUGA-3^′^ |
| miR-92a | Upstream primer:5^′^-ACAGGCCGGGACAAGTGCAATA-3^′^ |
